# Brief geriatric assessments for older adults in the community in Singapore: a policy brief

**DOI:** 10.3389/fpubh.2025.1584990

**Published:** 2025-08-06

**Authors:** Woan Shin Tan, Jonathan Gao, Ezra Ho, Lay Khoon Lau, Penny Lun, Yew Yoong Ding

**Affiliations:** ^1^Geriatric Education and Research Institute, Singapore, Singapore; ^2^Department of Geriatric Medicine, Tan Tock Seng Hospital, Singapore, Singapore

**Keywords:** brief geriatric assessment, community screening, community health, geriatric syndromes, stakeholder engagement, older adults

## Abstract

Early detection and assessment of geriatric syndromes and social issues is important to help older adults maintain health and functional ability. While comprehensive geriatric assessment (CGA) is considered the gold standard, it is resource intensive to implement. Shorter forms such as a brief geriatric assessment (BGA) could be an alternative. We adopted a multi-method, three-phase study to understand how BGAs could be implemented in the community. Findings suggest that BGAs can help to identify older adults with unmet needs or geriatric syndromes for further appropriate assessments. A BGA should include an assessment of physical health, psychological health, functional ability, mobility, and social needs. Stakeholder dialogues emphasised that BGAs should align with the existing system of screening and assessments spearheaded by other governmental agencies.

## 1 Background

The population of Singapore is ageing at an unprecedented rate. As of June 2024, 19.9% of the Singapore citizen population was aged 65 and above (1). Among older adults, the level of impairment in activities of daily living (ADL) and instrumental ADL is notably high, with a pooled prevalence of 23.6 and 39.3%, respectively (2). Similar trends are observed when we examined the prevalence of cognitive impairment (35.6%), sensory problems (32.3%), psychological issues (20.9%) and limitations in locomotion (20.8%) among community-dwelling older adults (3). By 2030, it is estimated that the proportion of older adults among Singapore citizens will grow to 24.1%, potentially resulting in a corresponding increase in the burden of age-related physical disability, functional and cognitive impairments, sarcopenia, and frailty (1). It is vital that health systems are designed to support the health and functional ability of older adults. This helps older adults live independently, reducing future burden on care services.

Early detection and assessment could signpost at-risk older adults to relevant preventive health services. However, functional declines and geriatric syndromes are often underdiagnosed as they do not fall into specific disease groups. Comprehensive Geriatric Assessments (CGA) have been shown to increase independence and reduce mortality risk among frail older adults in hospital settings (4). These holistic, multidimensional assessments support the identification of geriatric syndromes, geriatric conditions, psychosocial and functional issues, and support development of an integrated and coordinated care plan. Although evidence on its impact on health outcomes when carried out in community-based settings is still equivocal (5–7), many jurisdictions such as the United Kingdom (8) and Singapore have introduced community-based CGAs to provide person-centred holistic care to older adults.

In 2023, the Singapore Ministry of Health published the National Frailty Strategy Policy Report, which outlined recommendations to strengthen frailty prevention, detection, and management. The report recommended the conduct of CGAs performed by trained health professionals for older adults who are mildly frail with declines in intrinsic capacity as well as those who are moderately frail (9). This approach stratifies older adults based on frailty status and reserves CGAs for individuals who are most likely to benefit from them. However, the assessment itself is still time-consuming to carry out, and community-based care providers may face specific challenges in operationalising and optimising the delivery of a CGA (7).

In translating national policies into practice, it is often necessary to contextualise the guidance. In the Singapore context, private clinics attend to approximately 80% of all primary care admissions (10). However, public clinics (also known as ‘polyclinics’) bear a disproportionate burden of patients with complex conditions, such as non-communicable diseases because public patients can receive a comprehensive range of subsidised health services (11). Due to this imbalance between public and private healthcare utilisation, polyclinic doctors face long hours and heavy workloads, attending to approximately 40 patients per day (12, 13). In such busy settings, conducting a CGA is challenging as it may take more than an hour to complete the assessment. Additional time is also required to tailor recommended interventions to individuals’ needs. Shorter forms such as a brief geriatric assessment (BGA) can be an alternative. However, little is known about its assessment domains in comparison to CGAs, its role in clinical care, and implementability in Singapore. This policy brief syntheses the international evidence around BGAs, obtains local consensus, and identifies implementation considerations from health, social, and policy stakeholders to understand the feasibility of implementing BGAs in the community. Drawing lessons from the Singapore context, we highlight the potential for effective preventive health design in the community setting to enable healthy ageing among older adults.

## 2 Methods

**Figure 1:**
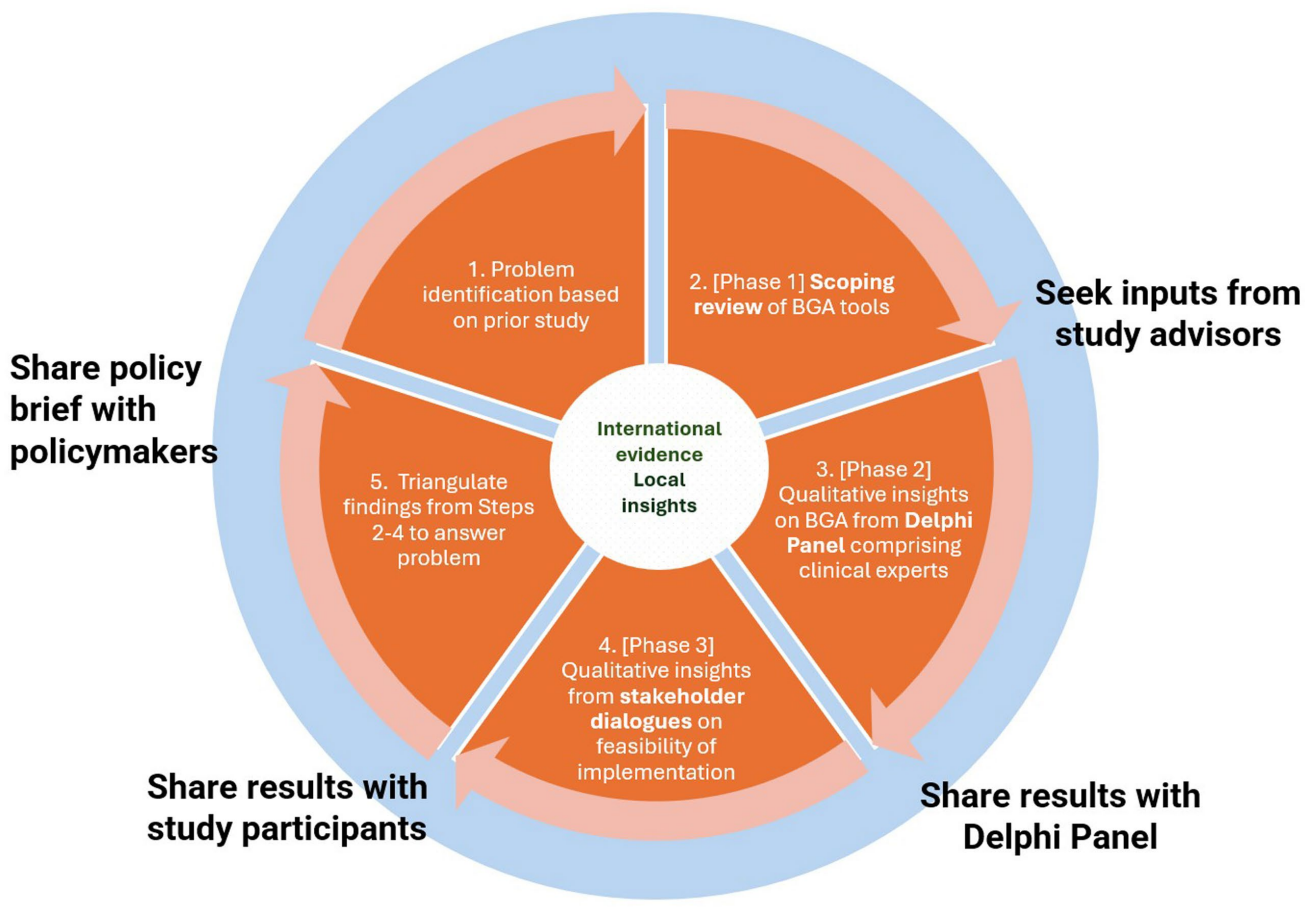
Approach to constructing the evidence for the policy brief.

A sequential multiple methods approach was adopted to mobilise a range of evidence across a three-phase study. Evidence from a scoping review was combined with local clinical experts’ opinion and qualitative findings from stakeholder dialogues, to consider options to address the problem, and key implementation considerations (14). A system of transparent and responsive engagement was cultivated with our stakeholders by sharing our findings with each relevant group. The final stage involved synthesising the findings into a policy brief (Figure 1).

In Phase 1, we conducted a scoping review of international literature to identify BGA tools that have been implemented or evaluated in primary care or community settings (15). The scoping review was carried out according to PRISMA guidelines. We used a Population-Concept-Context search strategy (older adults ≥ 60 y; ‘brief/abbreviated geriatric assessment’; community or primary care settings) to search CENTRAL, Pubmed, and Embase databases for relevant papers since inception—April 2024. We sought to identify the tools, commonly assessed domains, and the role of BGA in relation to care pathways in the community. This helped us understand how BGAs have been utilised and provided insights into the practical considerations of implementation. 25 studies examined the implementation and/or evaluation of BGA in community and primary care settings. This synthesis also highlighted gaps in knowledge and generated insights to inform policy recommendations.

In Phase 2, we drew on findings from our scoping review to design a two-round electronic Delphi study to understand the core requirements such as target population, essential assessment domains, as well as implementation considerations for a BGA in Singapore (16). Three expert geriatricians with clinical and research experience in geriatric assessments contributed to statement development. In total, 72 statements were developed to elicit experts’ perspectives on BGA design and implementation decisions. For example, statements on which domains should be covered by a BGA, and how it should be administered. These statements were sent to a purposive sample comprising geriatricians and family physicians from publicly funded healthcare provider groups in Singapore. Fifteen out of the 16 participants responded and participated in the study. An *a priori* dual criteria was used to define consensus as having at least 75% of the panel members disagreeing or agreeing to a statement and an interquartile range of one or less (17, 18).

In Phase 3, we conducted a series of dialogues to understand the perspectives of administrators and implementers working in health and community care on how a BGA fits in with the overall national and organisational strategy for the care of older adults, and to explore the feasibility of implementing a BGA in practice. Findings from our Delphi study were used to design our topic guides and guide conversations with dialogue participants. We adopted purposive sampling to obtain a range of perspectives from those who are involved in formulating frailty policy, and individuals responsible for the operationalisation of screening and assessment of older adults in the community. In total, we held discussions with 29 stakeholders across 14 organisations. Five stakeholders worked at national agencies, three stakeholders represented national healthcare organisations, seven stakeholders were from community nursing teams and polyclinics, 11 from social care organisations, and three general practitioners.

## 3 Implications from the evidence

### 3.1 Role of BGA and implied care pathway

**Figure 2:**
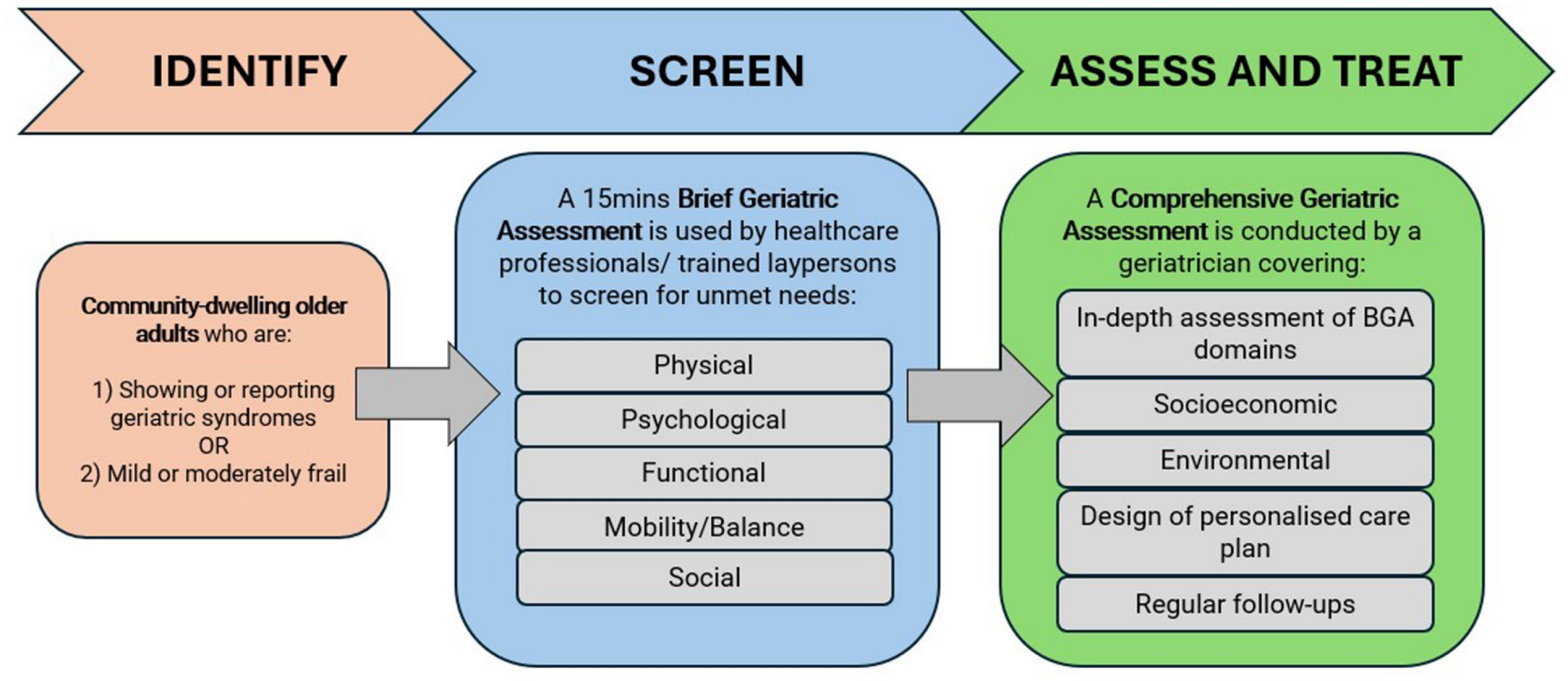
Role of BGA and implied care pathway.

Despite widespread views that CGAs are resource intensive to implement in community and primary care settings, our scoping review revealed that there were no valid alternatives to the CGA (15). Instead, brief assessments are typically used to identify older adults with unmet needs or geriatric syndromes to direct them to further assessments or referrals along the continuum of care. Moreover, there is a lack of evidence to support replacing a full CGA with a shortened version. Local clinical experts concurred with this. There was consensus amongst the 15-member Delphi panel on initiating a CGA as a confirmatory step following a screen-positive by a BGA, before follow-up care could be safely recommended (16). Based on the findings from the Delphi panel, Figure 2 shows the role of BGA and the implied care pathway for older adults in the community.

### 3.2 Target population

There was consensus among the Delphi panel that a BGA should target older adults showing or reporting geriatric syndromes, or older adults with mild to moderate frailty (16). Statements targeting broader groups such as those living alone, aged 75 and above, or receiving regular primary care services did not achieve consensus. While it could be simpler to implement a policy that uses a broad age cut-off or to target older adults attending primary care clinics, the panel preferred the use of a more targeted approach as it is operationally more efficient. In addition, individuals who are severely frail would not benefit from a brief assessment and these individuals should be referred for CGA instead.

### 3.3 Dimensions and mode of assessment

The scoping review highlighted that BGA tools commonly assessed the physical health, psychological or mental health, functional ability, and mobility or balance of the older person (15). In addition to these four domains, the Delphi panel strongly agreed that a BGA should also consider one’s social situation as living alone and not having any caregivers could negatively affect an older person’s health outcomes and quality of life (19, 20).

In the research literature, studies have relied on self- or proxy-reported accounts or made use of healthcare professionals or trained non-healthcare personnel to administer the assessment (15). For assessments to be sufficiently accurate for clinical use, there was consensus among the Delphi panel members that BGAs can be feasibly administered by healthcare professionals other than doctors (16). Although 73% of the panellists agreed that trained non-healthcare professionals could administer a BGA, consensus was not reached. However, there is a need to balance accuracy of assessment and implementability. Given current demands on staffing capacity, it is likely impractical to strictly adhere to using healthcare professionals. Establishing a system for training, audit, and collection of feedback could help build trust and confidence around the involvement of non-healthcare professionals for selected sections of a BGA.

### 3.4 Barriers to BGA implementation

There are currently no systematic screening procedures in place in the public and private primary care sectors. Assessments are typically conducted when older adults present with various complaints. A variety of different tools may be used by community nurses carrying out modified CGAs, and by day care and home care providers. Outside of healthcare, there is however an existing system of mass screening. This is led by the Silver Generation Office (SGO), a governmental agency that conducts outreach to older adults in the community. As part of the SGO’s community engagement work, trained volunteers identify vulnerable older adults by conducting door-to-door visits and administering a questionnaire that covers domains on an individual’s physical health, psychological health, functional abilities, balance, and social needs. The SGO provides Active Ageing Centres, which are responsible for keeping older adults physically, mentally and socially active, with the screening results for residents living within their neighbourhood. Given the wide outreach of SGO, there is a general perception that a BGA will be duplicative in the community sector.

Stakeholders also perceived the feasibility of implementing a BGA to be contingent upon the resolution of several systemic barriers including manpower and infrastructural constraints. Its adoption may be hampered by the lack of a clear national framework that links and guides the goal of universal and targeted screening across the care continuum. Stakeholders also emphasised the importance of ensuring adequate downstream capabilities and capacity to manage screened positive older adults, and the need to set up data-sharing and communications platforms between various stakeholder groups.

There were also concerns regarding the trustworthiness of BGA findings and its potential downstream impact. Stakeholders had reservations about the conduct of psychological and/or cognitive screening as part of a BGA due to a perceived lack of expertise to conduct such tests in community settings. Furthermore, stakeholders cautioned that community workers will need to carefully communicate and help older adults understand BGA results given the societal stigmas associated with being tested positive for certain health conditions.

**Figure 3:**
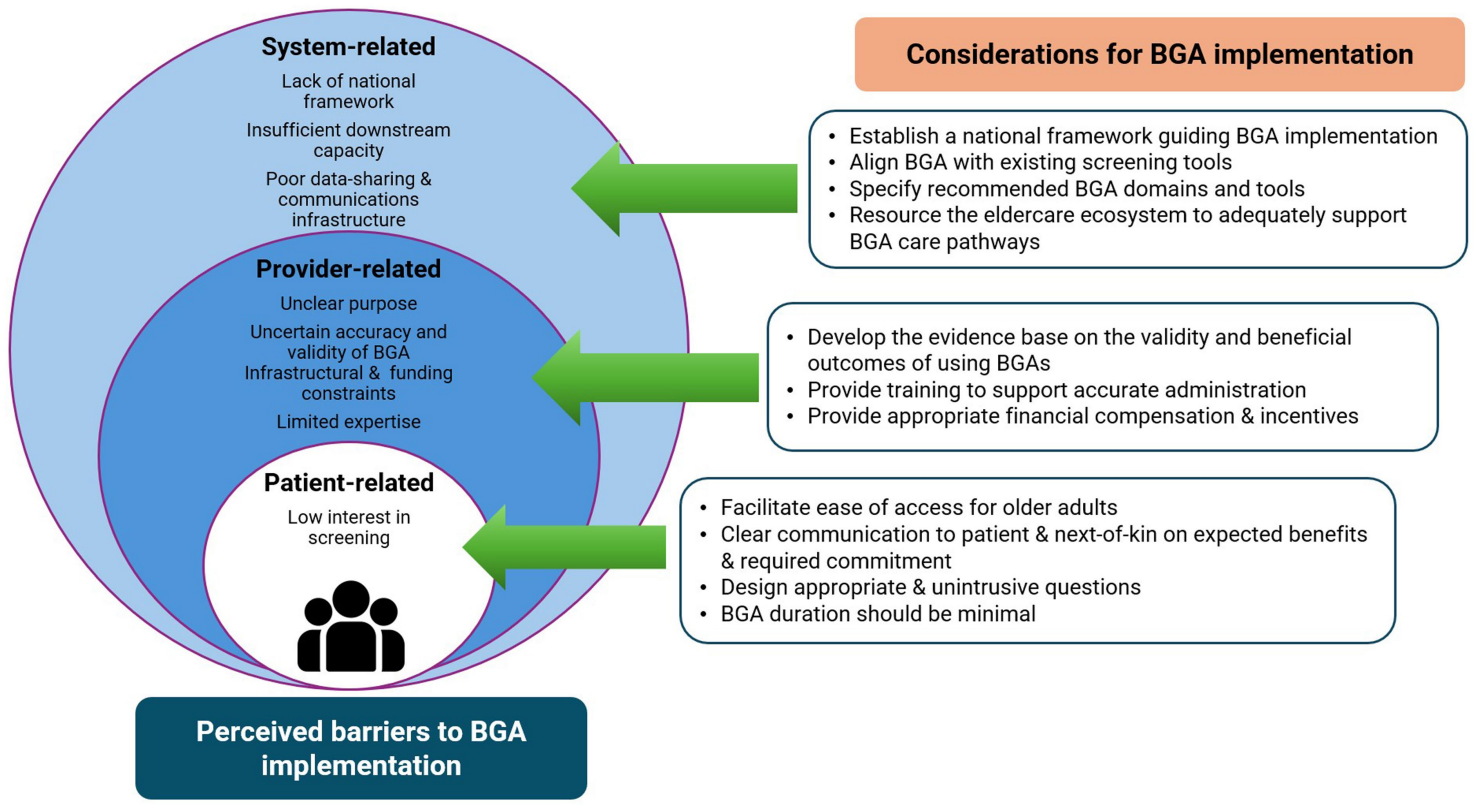
Perceived barriers and actionable recommendations.

In general, many stakeholders reported that older adults have low interest in participating in screening or health assessment activities as they may prefer to seek care only when they are experiencing symptoms or signs of an active disease. Additionally, the lack of information around the implications of the screening and expectations on follow-up recommendations may be disempowering when patients are uninformed or feel uncertain about making health decisions. Consequently, patients are less likely to take ownership of their health, contributing to low follow-through rates for screening and treatment. Stakeholders also expressed concerns over the appropriateness of allocating resources to screening, follow-up assessments and treatment when patients may be less receptive to following through (Figure 3).

## 4 Policy options and implications

Early screening and assessments can better detect and signpost at-risk seniors with geriatric syndromes and social issues to activities and services that could maintain and prevent further declines in their physical and mental reserves. However, a systems approach is needed to link the complex network of service provision in the community to create value for older adults. To overcome the barriers, the following policy options may better support the implementation of a BGA in the future. These policy options are complementary and ought to be implemented to collectively support older adults to age independently in their communities.

### 4.1 Establish care pathways

Identifying unmet health and social care needs in older adults is already part of our national community engagement effort. One policy option would be to establish evidence-based care pathways that spans social and healthcare sectors. However, given that few studies have examined the impact of BGA when combined with clinical intervention pathways, future research should seek to clarify longer-term impacts to inform policy, and to provide an evidence base to support its integration into routine care. It is also essential to establish clear indications for a CGA. If a CGA is not clinically necessary, or if patients prove to be unreceptive towards a full assessment, there should be management pathways available for specific conditions to guide their care (16).

### 4.2 Harmonise assessment tools and share data across settings

Given the high variability in health assessment tools used by various governmental agencies and care providers, one option is to commission a review of existing tools to harmonise the assessment dimensions, and to recommend a set of reliable and validated objective measures and/or self-reporting tools for use in practice. Establishing a standard set of validated measures will give providers greater confidence in the validity of the assessment results. Additionally, care pathways will become more efficient by reducing unnecessary duplication of assessments and tests. To share such data across health and social care settings, we ought to explore how to leverage the National Electronic Health Record (NEHR) system, which currently serves as the central repository of patients’ summarised health records across different healthcare providers.

### 4.3 Create a policy implementation blueprint

An eldercare ecosystem that meets the needs and preferences of older adults is foundational for frailty management at the population level. Linking the overall aims of preserving functional ability with evidence-based practices and resources can provide a roadmap on how to achieve the desired health outcomes for community-based older adults. This, however, cannot be achieved without adequate resources to support the various service lines and acceptance from older adults. Without timely matching of services to needs, early detection could create further stress on existing healthcare resources. Therefore, investment in the development of a national implementation blueprint for aged care defining the goals and objectives, roles of health and social care providers, and a resource and capacity projection plan, can increase the likelihood of effective policy implementation. This will require the formation of a multisectoral stakeholder group to contribute their views and expertise to develop, and act as sectoral champions to sustain its use in practice.

## 5 Actionable recommendations

Specific actions are needed to address barriers identified to hinder the implementation of BGAs. These recommendations aim to promote greater adoption of a BGA as part of a care pathway by primary and community care providers. In addition, it is crucial to understand the reasons for non-acceptance and to include strategies to address those specific barriers as part of the implementation blueprint.

### 5.1 Enhancing provider adoption of BGAs

The screening tool should be reliable and valid, as well as add value to care providers by improving work efficiency. Before widespread implementation, we would suggest pilot-testing the feasibility of administering and incorporating such tools within community-based clinics or centres. We recommend specifically testing the validity and predictive performance of potential BGA and CGA tools to ensure that the results are reliable and meet clinical standards for use to assure providers that professional standards of clinical safety and accountability are maintained. Furthermore, there needs to be adequate training for personnel administering the screening tool and policymakers should consider how to provide sufficient funding to incentivise community-based providers for the time taken to conduct the screening and subsequent assessments along the care pathway.

### 5.2 Meeting the needs and preferences of older adults

According to our research, older adults showed little interest in geriatric syndrome screening and management programmes. The benefits and the implication of a BGA need to be effectively communicated to patients and their family members. This may motivate greater participation in screening, assessment, or follow-up interventions. We recommend for the BGA to be designed such that it can be completed within a short period of time, and not be too intrusive to older adults. Older adults may reject BGAs and downstream interventions for a myriad of reasons. Tailored approaches intended to address the preferences and meet the needs of different groups of older adults are therefore crucial to improving their acceptance of a BGA.

## 6 Conclusion

Overall, our study suggests that BGAs can help to identify older adults with unmet needs or geriatric syndromes for further appropriate assessments. Our scoping review and electronic Delphi study suggest that a BGA should serve as a precursor to undergoing a CGA, and include an assessment of physical health, psychological health, functional ability, mobility, and social needs. Stakeholders emphasised that BGAs should align with the existing system of screening and assessments spearheaded by other governmental agencies. They highlighted the need for a national policy linking targeted screening with adequately resourced downstream care processes. Concerns about the psychometric properties of BGAs, their trustworthiness, and impact on downstream outcomes should be addressed. Finally, data linkages between health and social care providers should be strengthened through inter-sectoral rather than intra-sectoral information technology integration. A well-resourced and coordinated policy effort will be needed to effectively address these issues, improve the delivery of timely preventive care, and support healthy ageing among older adults in Singapore.
